# Body Positions and Physical Activity Levels Modulate the Ratio of Abdominal to Thoracic Breathing and Respiratory Rate in Young Individuals

**DOI:** 10.3390/jcm13247825

**Published:** 2024-12-21

**Authors:** Szonja Decker, Tamás Horváth, Johanna Takács, Akos Koller

**Affiliations:** 1 Research Center for Sports Physiology, Hungarian University of Sports Science, 1123 Budapest, Hungary; decker.szonja@gmail.com (S.D.); tom.horvath.md@gmail.com (T.H.); 2 Department of Morphology & Physiology, Faculty of Health Sciences, Semmelweis University, 1088 Budapest, Hungary; 3 Department of Social Sciences, Faculty of Health Sciences, Semmelweis University,1088 Budapest, Hungary; spss.stat@gmail.com; 4 Department of Physiology, New York Medical College, 15 Dana Rd, Valhalla, NY 10595, USA; 5 Translational Medicine, HUN-RES-SE, Cerebrovascular and Neurocognitive Disorders Research Group, Semmelweis University, 1085 Budapest, Hungary

**Keywords:** coronavirus disease (COVID-19), breathing mechanics, respiratory belts, respiratory rate, regular physical activity level

## Abstract

**Background/Objectives**: The COVID-19 pandemic highlighted that body positions substantially affected the mortality rate. We hypothesized that body position modulates the contribution of abdominal (AB) and thoracic breathing (TB) to the breathing cycle (BC), as well as respiratory rate (RR). In addition, we hypothesized that physical activity level can increase the contribution of abdominal breathing. **Methods**: Thus, we used plethysmography respiratory belts to measure changes in abdominal (AB) and thoracic (TB) circumferences, their ratio (AB/TB), and respiratory rate (RR) under resting conditions. Measurements were taken in four body positions—standing (St), sitting (Si), supine (Su), and prone (Pr)—for two groups of young adults (aged 21 ± 2 years) with different physical activity levels (low and high PA). **Results**: The AB/TB ratios significantly differed between the body positions (Si: 45.5/54.5%, St: 40.5/59.5%, Su: 56.8/43.2%, Pr: 52.2/47.8% (*p* < 0.001)). AB was significantly the highest in Su and lowest in Si and St (*p* < 0.001). There was a significant difference in respiratory rate (RR) between the four body positions (*p* = 0.005). RR in the four body positions was the following: Si: 15.1, St: 15.0, Su: 13.7, and Pr: 14.4. RR was the lowest in Su (13.7), where AB was the highest (56.8%), and RR was the highest in Si (15.1) and St (15.0), where TB was higher compared to Su (*p* < 0.001). PA significantly affected the various body positions’ AB/TB ratio and RR. The high PA group showed a significant difference in the AB/TB ratio between the body positions (*p* < 0.001). The low PA group showed a significant difference in RR between the body positions (*p* = 0.025). **Conclusions**: In young, healthy adults, we found that body position significantly influenced the abdominal/thoracic breathing ratio during the breathing cycle. The supine position showed the highest contribution of abdominal breathing, which correlated with the lowest respiratory rate. Additionally, a higher level of physical activity increased the contribution of abdominal breathing in the Si, Su, and St positions, suggesting a more energy-efficient breathing pattern. These findings suggest the potentials for breathing pattern monitoring and position-based interventions to improve healthcare outcomes and enhance sports performance and recovery.

## 1. Introduction

Being a vital function, investigating and monitoring breathing is essential during healthcare and physical activities (sports) to optimize its function [1]. Breathing is achieved by creating negative and positive pressures compared to the atmospheric pressure, which moves airflow in and out of the lungs [2,3,4]. Some literature distinguishes breathing into two main parts: abdominal and thoracic breathing (AB and TB). The movement of the diaphragm primarily drives abdominal breathing, whereas thoracic breathing also involves additional accessory respiratory muscles in the upper thoracic cage [2,5,6,7].

The diaphragm muscle is the primary muscle involved in breathing [8]. It is thought that abdominal breathing is more optimal than thoracic breathing, as the diaphragm is responsible for approximately 70% of the inhaled air volume during inspiration [9,10,11,12,13,14]. Usually, abdominal breathing is characterized by a slow, deep breathing pattern and synchronized movements of the abdomen and thorax [15,16].

In 2013, Pascal et al. demonstrated the positive effects of prone positioning in the treatment of acute respiratory distress syndrome (ARDS) [17], a finding that gained renewed importance during the coronavirus disease 2019 (COVID-19 pandemic) [18,19,20]. Indeed, the study published by Shelhamer showed that body positions have significantly affected mortality and morbidity, which were significantly lower for patients lying prone compared to those lying on their backs [18]. Several factors can alter the mechanics of breathing such as health conditions and various levels of physical activity [5,6,21,22,23].

Previous studies have confirmed that developing proper breathing mechanics not only positively impacts quality of life but also improves breathing in patients and enhances the performance of elite athletes [2,5,19,24,25,26].

### 1.1. Breathing Mechanics of Patients

In patients with impaired respiratory functions, such as those suffering from ARDS or COVID-19, specific breathing techniques are essential to improve respiratory efficiency and to enhance overall quality of life [17,18,27]. According to several studies, the COVID-19 pandemic advanced the respiratory field, as multiple strategies developed during this time have since been applied across various treatments [23,28,29].

For example, body positioning, such as prone positioning, is now commonly used to improve oxygen levels and manage respiratory issues in both patients and healthy individuals. Pulmonary function is significantly influenced by body position, though different conditions benefit from different specific positions [29].

Balban et al. observed the need for quick, cost-effective respiratory techniques during the COVID-19 pandemic to address the widespread health issues that were managed through breathing exercises. The pattern and depth of breathing have been shown to directly affect ventilation [26]. Additionally, breathing exercises enhance meditation, mental focus, cognitive function, and concentration—all of which are critical in sports, where mental and physical demands intersect and are both critical for maximal performance [23].

### 1.2. Breathing Mechanics in Athletes

During maximal exercise, inspiratory respiratory muscles consume approximately 16% of the body’s total oxygen availability. This underscores the significant effort required for breathing, even in healthy young individuals [30]. It has also been reported that inadequate breathing patterns can affect endurance and compromise movement efficiency, thus limiting athletic performance [31]. Furthermore, it has been well documented that practicing diaphragmatic or abdominal breathing can enhance the overall physical and mental state of athletes [25,32,33].

In the present study, we aimed to elucidate whether young individuals with higher activity levels naturally exhibit this “more efficient” breathing pattern. This is important because previous studies have highlighted the lack of comparative data on the physiological impacts of different breathing techniques, such as the pattern of breathing in various body positions having a physiological impact on ventilation [26]. Thus, the present study focused on how activity levels affect the ratio of abdominal and thoracic breathing patterns, an aspect not widely studied or highlighted before. We measured changes in these patterns and their associated respiratory rates using an inexpensive and portable device. This approach allowed us to observe and quantify differences between the two groups, offering insights into how activity levels influence these breathing patterns.

Based on the above, we hypothesized that, under resting conditions, the mechanics of breathing and respiratory rate would differ in various body positions due to the varying contributions of abdominal and thoracic breathing movements during the breathing cycle (BC). Thus, the present study aimed to observe these variations and statistically verify significant differences in breathing patterns based on the physical activity levels of healthy, young individuals. Specifically, we measured the abdominal and thoracic movements/circumferences and respiratory rates simultaneously in various body positions.

## 2. Materials and Methods

### 2.1. Study Sample

The present study was conducted on a sex-balanced, homogeneous sample of young, healthy adults aged 18–24 years (19 males and 18 females), all of whom were non-smokers with no reported cardiorespiratory or respiratory diseases. Participants were provided with verbal and written information about the measurements and signed a consent form, and their identities were kept anonymous. The research was approved by the Hungarian University of Physical Education and Sports Sciences Research Ethics Committee (approval number TE-KEB/13/2023).

The following data were collected: year of birth, sex, height, weight, thoracic and abdomen circumference in centimeters after maximum inspiration and expiration, type of sport practiced, and level of physical activity including hours/week of training.

According to the classification scheme of the European Society of Cardiology [34], physical activity levels can be categorized into three groups: recreational ≥ 4 h/week, competitive ≥ 6 h/week, and elite ≥ 10 h/week.

In the present study, in line with this classification, participants were divided into two groups based on their level of regular physical activity: a low activity level, ranging from 0 to 6 h/week (low PA, n = 15), and a high activity level, ranging from 7 to 10+ hours/week (high PA, n = 22). The low PA group had an average of 2.5 h/week of activity level, while the high PA group had an average of 9.3 h/week of activity level (Table 1).

### 2.2. Measurements and Calibration of Respiratory Belts

To assess abdominal and thoracic breathing, respiratory belts (ADInstruments TN1132/ST, Bella Vista, Australia) were utilized to measure the movement/expansion of the abdomen and thorax. The belts were connected to the ADInstruments Power Lab 4/30 data acquisition unit (ADInstruments, Bella Vista, Australia). The respiratory belts were positioned at the level of the tenth thoracic vertebra and the fourth lumbar vertebra. At these specific points, the expansions of the abdomen and thorax were measured in centimeters during maximal inhalation and exhalation. These two reference points were selected based on a review of previous studies and the morphology of the thorax and abdomen [5,6,24,35]. The movements of the lower ribs, specifically around the 7–10 thoracic vertebrae, create the greatest expansion during inhalation. Therefore, the respiratory belts were placed at the level of the ninth rib, which corresponded to the level of the tenth thoracic vertebra due to the rib inclination [36,37,38,39,40]. Respiratory belts were retightened before each new position measurement to account for potential loosening with changes in body position. In the prone position, the measuring sensors may have been influenced by body weight, which likely contributed to increased signal noise in this position.

Belt tension was adjusted to measure the maximum and minimum excursion. The respiratory belt can measure tension between 0 and 100 mV, so ideally the resting respiration oscillated around 50 mV, i.e., a slight tension was maintained. The belts were reset before fitting. As the abdomen and thorax/chest expand, the sensors tighten and deliver an electrical signal in mV linearly (proportionally) with the tightening. The respiratory belts were secured with adhesive tape on the subject’s skin and after each change in body position, the belts were readjusted if required. The ambient temperature of the examination room was maintained at 21 degrees Celsius with normal humidity.

Cardiovascular screening involved heart rate measurement and blood pressure assessment, with pulse oximetry placed on each participant’s index finger. To measure blood pressure and heart rate, an Omron Hem-907 (Omron, Kyoto, Japan) blood pressure monitor was used. Oxygen saturation (SpO2) was measured by pulse oximetry (Beurer PO45). Since all participants presented normal values, no additional cardiovascular tests were required. Obesity was monitored as a marker for potential metabolic conditions through BMI calculations, as it is known to impact respiratory function [41]. All participants were below the criteria for obesity based on BMI classification. Participants received both verbal and written explanations about the measurements, signed consent forms, and remained anonymous throughout the study. They were instructed not to eat for 4 h before measurements, though hydration was not restricted. Participants were also advised not to come directly from training, and they were seated for 10 min before any intervention or measurements began. Participants were encouraged to maintain their usual diet and drinking habits.

### 2.3. Four Body Positions

To elucidate the potential effects of body positions on breathing mechanics, we designed four different positions and uniquely measured changes in both abdominal and thoracic movements/expansions simultaneously, with two respiratory belts used to record changes in abdominal and thoracic circumference. Between various body positions, a one-minute rest period was kept with each measurement lasting ~20 min.

(1) Sitting position (Si): Participants’ feet were placed flat on the ground, with a 90-degree angle between the thighs and lower legs. They kept their spine straight without touching the back of the chair, as back support can influence breathing patterns [22].
(2) Standing position (St): Participants were in the standard anatomical position. The head and torso were upright. The arms were at the sides of the torso with the shoulders in neutral rotation, elbows extended, and palms facing forward. The fingers were also extended [42].
(3) Supine position (Su): the participant’s limbs were extended, feet close together, arms alongside the body, and the face was directed toward the ceiling.
(4) Prone position (Pr): Participants were allowed to turn their heads to the side for comfort.

In both prone and supine positions, the bed was horizontal, and no objects were used to elevate any body parts.

**Figure 1:**
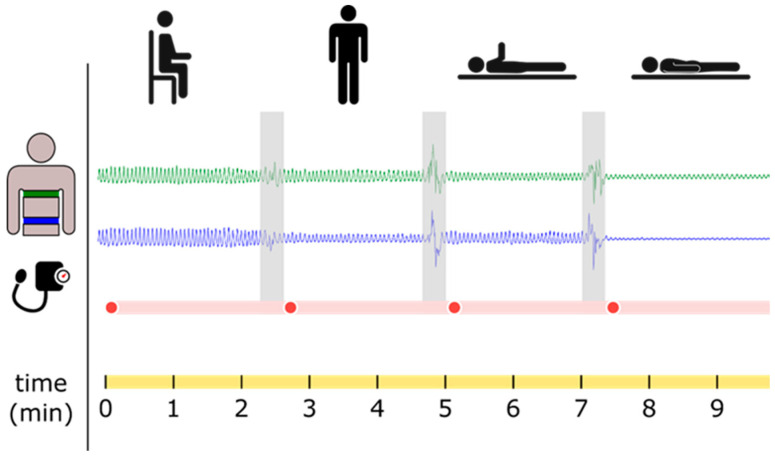
Experimental protocol: schematic diagram of the four body positions, as well as the corresponding respiratory belt signals, and systemic blood pressure, in resting conditions. The thoracic belt was positioned at the rib level where the greatest expansion was expected. The thoracic belt and the abdominal belt were aligned with the vertebral positions (Th10 and L4) to avoid any influence from anatomical differences between genders.

During the measurements, participants were asked to breathe calmly through their noses for one to two minutes in each of the following positions: sitting (Si), standing (St), supine (Su), and prone (Pr), as illustrated in Figure 1. An appropriate marker (e.g., “supine”, “sitting”, “standing”, “prone”) indicated the start of each body position. A one-minute rest period was provided between positions, with each measurement lasting ~25 min, during which two respiratory belts recorded changes in abdominal and thoracic circumference.

### 2.4. Data Recording and Signal Processing

Recording: Thoracic and abdominal breathing signals were recorded using an ADInstruments PowerLab 4/30 data logger with a 1 kHz sampling frequency. Data were processed in ADInstruments LabChart software (version 8, ADInstruments, Bella Vista, Australia) and exported in text format. Custom Python (Python 3.12.2, Wilmington, DE, USA) scripts were used for further analysis of these segments according to specific body positions.

Digital Filtering: a 4th order Butterworth band-pass filter was used in the 0.9–1.5 Hz range.

Signal Segmentation: Following filtering, within each breathing cycle, the maximum and minimum points were identified. Respiratory amplitudes for both abdominal and thoracic expansions were calculated as the difference between maximum and minimum values. The sum of the abdominal and thoracic breathing movements/expansions was obtained.

Amplitude Representation: Amplitude values indicated the relative contribution of thoracic and abdominal breathing to the sum. For each body position, mean and standard deviation of the relative contributions were calculated.

### 2.5. Statistical Analysis

Descriptive statistics were reported in mean, standard deviation, and relative frequencies. For the comparison of the four body positions, repeated measures analysis of variance (ANOVA) was used with Bonferroni confidence interval adjustment to compare main effects, and partial eta squared was calculated as effect size measurement. The two physical activity groups (low vs. high PA) were compared using independent samples t-test with Hedges’ g effect size measurement. To determine the effect of physical activity level on the difference between the four body positions, mixed ANOVA was conducted with simple main effects analysis. The level of significance was set before conducting the study to avoid p-hacking, and alpha was set at 0.05. For all statistical analyses, the level of significance was set at 0.05. Statistical analyses were conducted using IBM SPSS Statistics for Windows, Version 25.0 (IBM Corp. Released 2017, Armonk, NY, USA).

## 3. Results

Demographics and anthropometric data of the participants (n = 37) are shown in Table 1.

### 3.1. Comparison of the Abdominal to Thoracic Breathing Movement Ratios in the Four Body Positions

**Figure 2:**
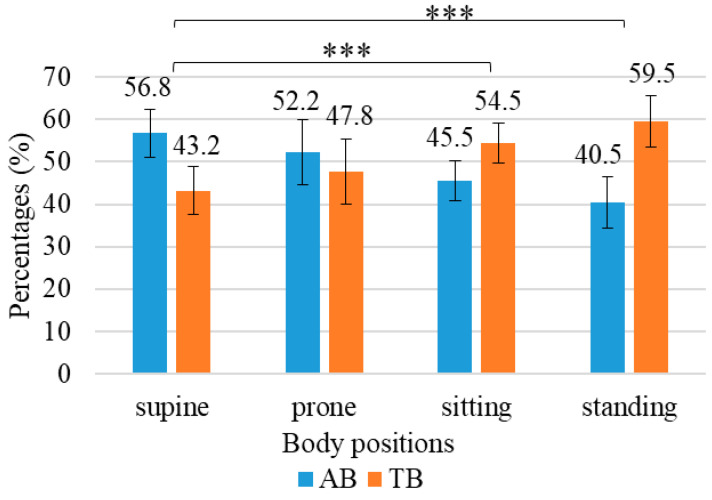
The figure shows the contribution (%) of abdominal (AB) and thoracic (TB) breathing movements to the breathing cycle in the four body positions. The contribution of AB was significantly greater in the supine position compared to sitting and standing positions. AB, abdominal breathing; TB, thoracic breathing; error bar, 95% confidence interval; ***, *p* < 0.001.

There was a significant difference between the four body positions (standing, St; sitting, Si; supine, Su; prone, Pr) in the ratio of abdominal and thoracic breathing movements (F(3,108) = 8.796, *p* < 0.001, η^2^_p_ = 0.20). Based on the main effects analysis, the AB/TB ratio was the highest in Su (56.8 [51.1, 62.4]/43.2 [37.6, 48.9]); it was significantly lower in Si (45.5 [40.8, 50.2]/54.5 [49.8, 59.2], difference [95%CI] = 11.3 [4.7, 17.9]) and St (40.5 [34.4, 46.6]/59.5 [53.4, 65.6], difference [95%CI] = 16.3 [8.9, 23.7]) (*p* < 0.001). The AB/TB ratio in Pr (52.2 [44.5, 59.9]/47.8 [40.1, 55.5]) did not reveal a significant difference compared to the other positions (Si, *p* = 0.696, difference [95%CI] = 6.8 [−5.0, 18.5]; St, *p* = 0.092, difference [95%CI] = 11.7 [−1.1, 24.6]; Su, *p* = 1.000, difference [95%CI] = −4.6 [−15.8, 6.6]) (Figure 2).

### 3.2. Comparison of Respiratory Rate in the Four Body Positions

**Figure 3:**
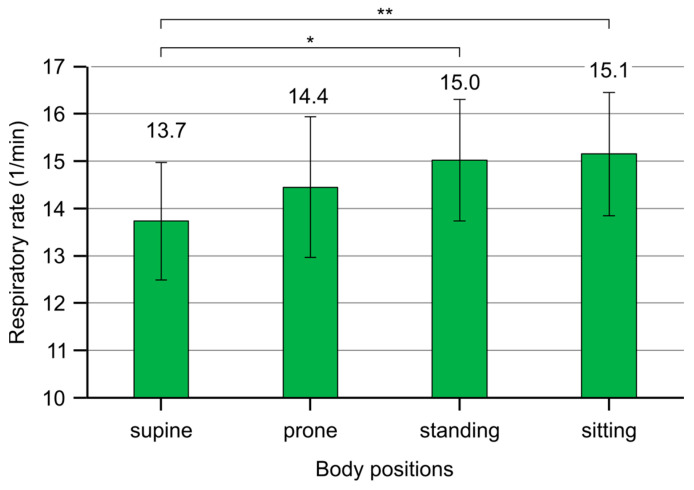
Respiratory rate (RR, 1/min) in the four body positions shows that RR significantly decreased in the supine position compared to sitting and standing positions. Error bar, 95% confidence interval; *, *p* < 0.05; **, *p* < 0.01.

There was a significant difference in respiratory rate (RR) between the four body positions (F(3,108) = 4.491, *p* = 0.005, η^2^_p_ = 0.11). Based on the main effects analysis, RR was significantly higher in Si (15.1 [13.8, 16.4], difference [95%CI] = −1.4 [−2.5, −0.4]) and St (15.0 [13.7, 16.3], difference [95%CI] = −1.3 [−2.5, −0.1]) compared to Su (13.7 [12.5, 15.0]) (*p* < 0.001). RR in Pr (14.4 [13.0, 15.9]) reveals a non-significant difference compared to the other body positions (Si, *p* = 0.824, difference [95%CI] = −0.7 [−2.0, 0.6]; St, *p* = 1.000, difference [95%CI] = −0.6 [−1.9, 0.8]; Su, *p* = 0.688, difference [95%CI] = 0.7 [−0.5, 2.0]) (Figure 3).

**Figure 4:**
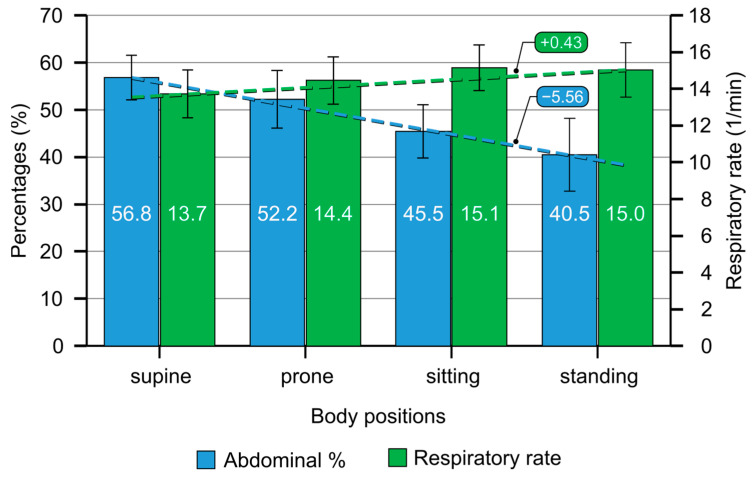
The figure shows changes in respiratory rates (RR, 1/min) as a function of body positions, showing a small increase in RR with a decrease from supine–prone–sitting–standing positions.

Next, we plotted the body position and respiratory rates showing a small increase in RR with a decrease from supine–prone–sitting–standing positions (Figure 4).

The text box bubbles show the average difference between the sequentially arranged groups (approximate slope). For example, the respiratory rate increased by 0.43 breaths per minute between the groups, and the abdominal breathing contribution decreased by 5.56%. Error bar: 95% confidence interval.

### 3.3. The Effect of Physical Activity on the Ratio of Abdominal and Thoracic Breathing Movements

**Figure 5:**
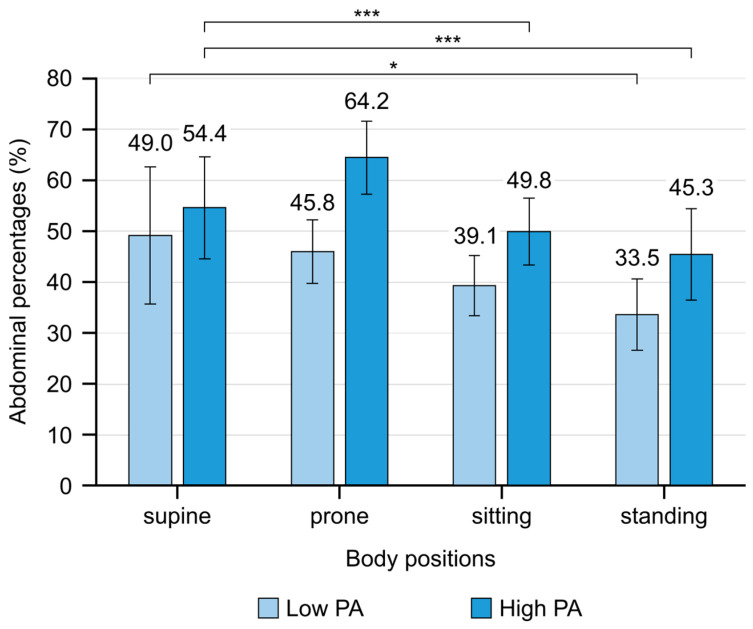
The percentage contribution of abdominal (AB) breathing movements in the four body positions in the low PA and high PA groups. In the low PA group, the contribution of AB was significantly higher in the supine position than in the standing position. In the high PA group, the contribution of AB was significantly higher in the supine position compared to sitting and standing positions. (AB, abdominal breathing; error bar, 95% confidence interval; ***, *p* < 0.001; *, *p* < 0.05.)

Regular physical activity level had a significant effect on the AB/TB ratio between the various body positions, revealing a significant difference in AB/TB ratio between the body positions in the high PA group (F(3,63) = 6.595, *p* < 0.001, η^2^_p_ = 0.24). The AB/TB ratio was significantly higher in Su and the lowest in St and Si (*p* < 0.001). Pr did not differ from the other body positions. In sum, the low PA group showed a non-significant difference in the AB/TB ratio between the body positions (F(3,42) = 3.394, *p* = 0.055, η^2^_p_ = 0.20). Based on the pairwise comparisons, the AB/TB ratio was significantly higher in Su compared to St (*p* = 0.045) (Figure 5).

### 3.4. Effects of Regular Physical Activity Level on Respiratory Rate

**Figure 6:**
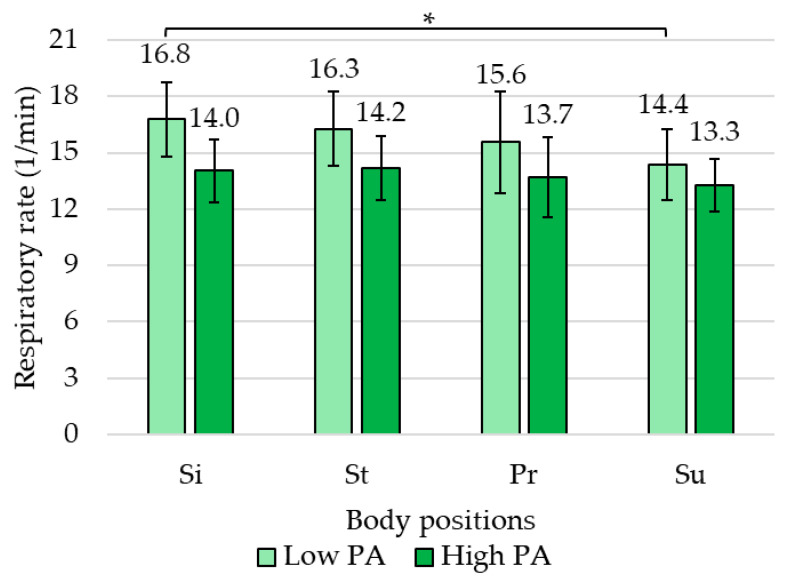
Respiratory rate (RR, 1/min) in the four body positions. Low PA (light green) and high PA (dark green) groups. In the low PA group, RR was significantly higher in sitting than in the supine position. In the high PA group, RR did not show a significant difference between the body positions. *, *p* < 0.05; error bar, 95% confidence interval.

Regular physical activity level also had a significant effect on RR between the various body positions, and the low PA group showed a significant difference in RR between the body positions (F(3,42) = 3.446, *p* = 0.025, η^2^_p_ = 0.20). The RR was significantly higher in Si than in Su (*p* = 0.014). The high PA group showed a non-significant difference in RR between the body positions (F(3,63) = 1.332, *p* = 0.272, η^2^_p_ = 0.06) (Figure 6).

Finally, the difference between low and high PA groups was examined in each body position. In the case of the AB/TB ratio, Si, St, and Su showed a significant difference with a higher AB/lower TB in the high PA group compared to the low PA group. In Pr, the two PA groups did not show significant differences in AB/TB ratio (Table 2). In the case of RR, Si showed a significant difference with a higher RR in the low PA group compared to the high PA group.

In the other body positions, the two PA groups did not show significant differences in RR (Table 2).

## 4. Discussion

In the present study, we investigated the contribution of abdominal and thoracic breathing movements (breathing patterns) to the breathing cycle in young, apparently healthy adults in four different body positions.

The salient findings of the present study are the following:

(1) The contribution of abdominal and thoracic breathing movements to the breathing cycle differed significantly in the four body positions. Importantly, the highest abdominal breathing movement occurred in the supine position, while the highest thoracic breathing occurred in the standing and sitting position.
(2) Correspondingly, the respiratory rate was lowest in the supine position and highest in the sitting and standing positions.
(3) Furthermore, a higher level of regular physical activity was associated with an increased contribution of abdominal breathing movements in the sitting, standing, and supine body positions.

### 4.1. Abdominal vs. Thoracic Breathing

The present study was prompted by the global COVID-19 pandemic, which raised several questions regarding the efficacy and functioning of the respiratory system in different body positions. This study contributes and adds to the findings of prior research on abdominal and thoracic breathing patterns. Previous studies suggest that predominant abdominal breathing is a healthier pattern, as it promotes better thoracoabdominal synchronization and allows for greater air volume movement [5]. It is more economical in terms of mechanical work and thus requires less energy [2,5,24]. Additionally, abdominal breathing can modulate respiratory rates [2,5,23,24], which may be important in the management of diseases and other conditions. Indeed, Kayser et al. showed that changes in respiratory rate can indicate several underlying health problems. Thus, it is important to monitor respiratory rates not only in patients but also in healthy individuals and athletes as well [43,44].

*Other aspects of abdominal breathing*

1. *Efficiency of abdominal breathing*: Abdominal breathing is more energy-efficient, using less energy for respiration and allowing more energy for the activity of other skeletal muscles. Abdominal breathing increases the pressure difference, moving the air from outside into the lungs, allowing for a larger intake of air and oxygen while maintaining lower respiratory rates, which helps reduce overall respiratory effort [2,5,23,24].
2. *Abdominal breathing and venous return*: Abdominal breathing, coupled with a lower respiratory rate, can enhance venous return due to the increased efficiency of the respiratory pump [23]. Previous studies have emphasized that respiratory rate plays a crucial role during exercise, where it becomes particularly important for maintaining circulation and oxygen delivery [2,5,23,24]. These findings are consistent with the results of the present study, where individuals with higher levels of physical activity demonstrated more predominant abdominal breathing movements and lower respiratory rates (Figure 5 and Figure 6).
3. *The central role of diaphragm*: Breathing is regulated by a complex neural network, with the diaphragm serving as the primary muscle responsible for generating pressure during the process [8,45]. With the control of the diaphragm, the lower ribs remain down and expand laterally, allowing the abdomen to expand [2,7,46]. The greater the difference between the inhalation and exhalation positions of the diaphragm, the greater the pressure difference (in and out) and therefore the greater the tidal volume. A stronger contribution of abdominal/diaphragmatic breathing likely results in an increasing tidal volume and a decreasing respiratory rate, indicating a more efficient breathing pattern [2].

Abdominal breathing has many benefits in patients (e.g., COPD and asthma), healthy individuals, and athletes alike, as it increases blood oxygen levels, increases tidal volume, enhances lung clearance, increases venous blood flow, improves posture, and reduces blood pressure, heart rate, stress, and anxiety [2,5,16,47,48].

### 4.2. Body Positions Change the Ratio of Abdominal and Thoracic Breathing

Body positions had a significant effect on the contribution ratio of abdominal and thoracic breathing (Figure 2). Our results indicate that abdominal breathing movements were significantly the highest in the supine position (56.8%) and lowest in the standing (40.5%) and sitting (45.5%) positions. Therefore, thoracic breathing was the highest in the standing (59.5%) and sitting (54.5%) positions and lowest in the supine position (43.2%). In the prone position, a similar ratio of abdominal and thoracic breathing movements was seen (AB/TB: 52.2/47.8%).

During the COVID-19 pandemic, prone positioning became important, although its effectiveness in acute respiratory distress syndrome had been studied earlier, because it significantly reduced mortality and improved patients’ respiratory parameters [49,50]. In the present study, we found that in the supine position, the proportion of abdominal movement was higher compared to the prone position, where the AB/TB ratios were nearly similar.

### 4.3. Factors Affecting the Mechanics of Breathing

Body position has a significant effect on breathing [13,17,51,52,53,54,55]. Different body positions affect the contribution of abdominal and thoracic breathing: vertical positions, such as sitting or standing, primarily increase thoracic breathing, whereas horizontal positions, such as supine, increase abdominal contribution during resting breathing [13,22,55,56,57]. The increase in the percentage of abdominal breathing movement from sitting to supine can be partially attributed to the elastic properties of the abdominal wall, the diaphragm’s role in maintaining postural stability, and the influence of gravity [22,54,55].

Posture also affects the function of the respiratory muscles. While upright, the diaphragm supports both respiration and postural stability, but this need for postural stability diminishes in the supine position, contributing to the predominance of abdominal breathing [58,59,60,61].

Several interesting considerations can be made regarding factors that may also affect breathing patterns in different body positions.

*(a) Gravity*: In the standing position, ventilation and perfusion are generally higher at the lung bases because of gravity, creating a close-to-ideal ventilation–perfusion (V/Q) match in the middle/lower regions. Positioning affects V/Q distribution: supine positioning can decrease lung capacity and worsen V/Q matching, while prone positioning promotes uniform gas exchange, beneficial for acute lung injury [62,63,64]. Evidence shows gravity explains only about 25% of blood flow variation, as lung structure plays a larger role, shaping V/Q distribution based on position and anatomical branching patterns [62].

The prone position under hyper-gravity enhances V/Q matching by preserving lung volume, evenly distributing blood flow, and improving oxygen saturation. In contrast, hyper-gravity in the supine position increases hydrostatic pressure, compressing the lungs [64].

*(b) Zone of apposition*: According to Boyle et al., one factor often overlooked by physical therapists is maintaining the optimal diaphragm appositional zone (ZOA). The ZOA refers to the area where the diaphragm meets the rib cage, particularly the inner surface of the lower ribs. During normal, relaxed breathing, in a standing position, the ZOA typically constitutes about a quarter to a third of the rib cage area. ZOA is thought to contribute to diaphragm function and trunk stability, as its reduction can lead to adaptive breathing strategies [54,58,65]. Furthermore, research shows that when the spine is flexed, the zone of apposition is more optimal [58], resulting in better mechanics of ventilation/breathing [25].

### 4.4. Respiratory Rate

One of the indicators of breathing efficiency is the respiratory rate [2]. Therefore, we investigated whether the respiratory rate changes across different body positions as the proportion of abdominal and thoracic breathing varies. Changes in respiratory rate showed significant differences between the body positions in the entire sample: in the supine position, the respiratory rate was significantly lower compared to sitting and standing positions. Given that there are thousands of breaths taken daily, this can be important from an energy consumption perspective. Indeed, the literature highlights that abdominal breathing is an energetically more efficient breathing pattern, which reduces respiratory rate and increases tidal volume [2,5].

One can expect the lowest respiratory rate in the supine position because it had the highest proportion of abdominal breathing. Accordingly, the lowest respiratory rate was measured in the supine position (13.7 breaths/minute), while the highest respiratory rates were measured in the sitting and standing positions (15.1/15.0 breaths/minute). This suggests that the supine position may be the most suitable for recovery after high-intensity physical activity to achieve optimal gas exchange.

It can be hypothesized that in different body positions, the respiratory pump supports venous return in varying ways [66,67,68], which could be a subject of future research. These results support the notion that abdominal breathing is a more economical form of breathing. It is important to consider that these results were measured at rest, and significant differences are likely to be present during various levels of physical activity.

### 4.5. Effect of Physical Activity Level on Breathing Mechanics

Various sports disciplines require specific breathing techniques due to the nature of the sport. In recent years, studies have been conducted across various sports to identify which breathing techniques can enhance athletic performance [15,20,23,33]. It has been observed that athletes have more economical resting breathing patterns compared to the general population, characterized by higher tidal volumes and lower respiratory rates [15,23]. Additionally, diaphragmatic/abdominal breathing has been shown to reduce the energy expenditure required for breathing [2,5]. Increased diaphragmatic action enhances endurance and supports high-intensity physical activity, as the diaphragm is more resistant to fatigue than the intercostal muscles [69].

Despite these facts being known, limited research has addressed the interaction between activity levels and respiratory mechanics in athletes. No studies were found that measured how physical activity affects the pattern of resting breathing. In the present study, participants were divided into two groups based on the level of their regular physical activity: high physical activity and low physical activity.

When looking at the differences between positions, the results in Section 3.3 (AB/TB) and Section 3.4 (RR) show that abdominal and thoracic breathing movements differ between the supine, standing, and sitting positions, especially in the high PA (physical activity) group. Respiratory rate (RR) was significantly higher in the sitting position compared to the supine position but only in the low PA group. However, when comparing the two groups (low and high physical activity groups), Table 2 shows that there are significant differences between the low and high groups in terms of AB/TB ratios in the sitting, standing, and supine positions, whereas the respiratory rate (RR) only differs in the sitting position.

Previous studies have demonstrated that athletes with higher physical activity levels tend to exhibit improved respiratory function, partly due to enhanced breathing mechanics. This adaptation helps meet the increased oxygen demands during aerobic and endurance exercises [23,25,70,71]. Driven by chronic stress on the respiratory system, these adaptations result in athletes displaying lower resting respiratory rates, higher tidal volumes, and the predominant use of diaphragmatic breathing, a pattern associated with greater respiratory efficiency [23,25,70,71]. Furthermore, lower respiratory rates, which are commonly observed in athletes, are linked to decreased resting heart rates, lower blood pressure, and improved blood oxygenation [32]. Nevertheless, in addition to breathing mechanics, the size of the lungs and the surface of alveoli are also important in gas exchange, which could be taken into consideration in future studies.

### 4.6. Applications of Our Findings in Health, Disease, and Exercise and Future

At rest, the energy required for breathing in healthy adults is about 2.4 joules per minute [72], representing a small oxygen cost, approximately 2% of total oxygen consumption (V^·^ O2) [73]. During intense exercise, however, this demand rises sharply. In untrained individuals, respiratory muscles use around 10% of the total oxygen uptake, while in highly trained individuals, this increases to 15–16% (equivalent to 300–500 mL/min). For individuals with chronic obstructive pulmonary disease (COPD), the oxygen cost can escalate to 35–40% of total oxygen consumption, indicating a much higher workload for the respiratory muscles [74]. This demonstrates that while the energy needed for breathing at rest is minimal, exercise and certain health conditions considerably increase the work of breathing. Additionally, it has been emphasized that the “choice” of breathing patterns must consider the mechanical properties of the respiratory system to keep the ventilatory cost to a minimum [73].

*(a) Applications of our findings for healthy individuals*: 

Integrating routine respiratory pattern screening yearly could serve as a preventive tool for identifying respiratory dysfunctions, due to physical and/or mental stress, postural abnormalities, and potential conditions such as chronic obstructive pulmonary disease (COPD). Such screening could be a low-cost, non-invasive intervention, allowing early diagnosis, prevention, and interventions before structural issues or diseases develop.

*(b) Applications of our findings to healthcare:*

*Several aspects can be mentioned regarding the importance of body positions and breathing patterns.*

Respiratory pattern monitoring: Standardizing respiratory screening for clinical care could allow early recognition of dysfunctional breathing patterns. Body positioning, combined with monitoring tools like respiratory belts, might improve outcomes in respiratory care by identifying deviations in breathing patterns.

Position-based interventions: the importance of body positioning observed in COVID-19 care suggests similar applications in other conditions [18,19,20].

Monitoring respiratory mechanics together with that of SpO_2_ and heart rate could improve patient outcomes, especially for those prone to respiratory compromise.

Another condition where breathing mechanics can be important is in the case of hemidiaphragm paralysis due to post-surgical or other reasons. Respiratory belts could potentially be used to assess unilateral hemidiaphragm paralysis and investigate how respiratory mechanics and efficiency may change in different positions in this condition [75].

*(c) Applications of our findings to exercise and sport*: 

Body position in exercise: implementing specific body positions before, during, or after exercise training may optimize breathing efficiency and improve performance.

Body position in training: breathing patterns vary across different sports (e.g., swimming, weightlifting, running, diving) for various reasons, including differences in respiratory rate and the ratio between abdominal and thoracic breathing [23,53,76,77,78,79]. Therefore, training may be optimized by aligning body position with the desired breathing pattern, allowing athletes to practice breathing techniques that support the specific respiratory demands of their sport.

Monitoring breathing patterns: Regular monitoring of an athlete’s breathing type (abdominal or thoracic) during rest could help detect and address dysfunctions that might hinder performance. The findings of the present study show that people with higher levels of physical activity have dominant abdominal breathing (Figure 5), suggesting the use of training methods or breathing exercises to support this pattern.

Recovering after strenuous exercise/sport: It is commonly observed that athletes assume a “hand and knee” position after exhausting sports activities to “catch their breath”. Indeed, this position may improve breathing mechanics by involving not only the primary respiratory muscles but also incorporating other accessory muscles. When hands press against a stable surface, certain muscles may further assist with breathing. For instance, the serratus anterior can contribute to respiratory effort, as changes in muscle length and the positions of origin and insertion alter its function [80]. This combination of diaphragmatic and accessory muscle engagement potentially leads to faster recovery. Indeed, “hands-on knees” and “hands-on head” positions showed significantly faster recovery of RR between interval training [25,81].

*(d) Future directions for investigation of breathing patterns in various conditions*: 

The findings of the present study suggest that breathing patterns and respiratory rate can change in various body positions even in resting conditions and likely change more dynamically and substantially under various conditions like exercise, stress, or disease. Future studies should focus on these dynamic conditions to increase our understanding of breathing patterns in terms of respiratory mechanics and kinematics. Future studies could also start implementing the preventive use of healthy breathing patterns. The findings of the present study underscore the significant impact of body positioning on respiratory mechanics, with applications in wellness, healthcare, and sports.

### 4.7. Limitations of This Study

To advance future investigations, several limitations and observations can be mentioned:

(a) In the present study, young, healthy subjects with a balanced sex distribution were intentionally selected to eliminate confounding factors that could affect the interpretation of the results. The tightly controlled parameters ensured that the sample size was appropriate for statistical analysis. However, it could be important for future studies to expand the investigation to include other age groups, individuals with various disease conditions, different body mass indices, and athletes from diverse sports to explore how breathing patterns may differ and to determine whether modifying breathing patterns could improve their respiratory function.
(b) Previous literature has mentioned that respiratory belts may loosen during changes in body position, requiring retightening each time before measurement can continue, which we tried to accomplish [82]. Thus, during the experiments, we checked the tightness of the belt in each position. We monitored the respiratory belts by observing the real-time respiratory amplitudes on the screen; if the amplitudes flattened after a position change, this indicated that the belts had loosened. In addition, in the prone position, measuring sensors may be affected by body weight, which is likely responsible for the greater signal noise in this position. Thus, in future studies, the respiratory belts should be repositioned to the back in the prone position.
(c) In the present study, we aimed to utilize a cost-effective and mobile method, and there is more precise, non-portable equipment like optoelectronic plethysmography (OEP) or portable respiratory vests available, but they have their limitations as well.
(d) In future studies, more attention should be given to avoiding any interference from coffee or energy drinks.

### 4.8. Future Potential of Body Positions and Exercise in Improving Breathing Efficiency

Although it is tempting to speculate, our study was conducted with young, healthy subjects, so we must be cautious in extrapolating our findings to diseased conditions, elderly individuals, or elite athletes. Nevertheless, continuing this investigation in other populations could provide more definitive insights regarding body positions in various pulmonary diseases, such as COPD and asthma, and in different sports and recovery settings. Now that we have observed substantial differences between low and high activity levels in breathing patterns, future studies could focus on specific sports categories. It is well documented that different sports require distinct breathing techniques based on the nature of the activity. For example, swimmers may need different breathing strategies due to water immersion [83], while runners’ breathing patterns may vary depending on whether they are sprinting or running long distances [23,53,77]. Weightlifters often use the Valsalva maneuver to create intra-abdominal pressure during heavy lifts [79]. Deep divers rely on specific techniques throughout a dive [78,84]. We believe that avenues for further research can build on these current findings to explore exercise physiology in greater depth.

## 5. Conclusions

The present study, by implementing an innovative and simple method for measuring abdominal and thoracic breathing movements (breathing patterns) using respiratory belts placed at specific points on the abdomen and thorax, provided several novel findings:

(1) In resting conditions, we found that body position significantly influenced breathing patterns in young, healthy adults, affecting the ratio of abdominal to thoracic breathing movements during the breathing cycle. The supine position showed the highest contribution of abdominal breathing movements, which related to the lowest respiratory rate, while the standing and sitting positions had the highest contribution of thoracic breathing movements and the highest respiratory rate.
(2) In the high physical activity level group, the abdominal and thoracic breathing movements differed between the supine, standing, and sitting positions.
(3) In the low physical activity level group, the respiratory rate (RR) was significantly higher in the sitting position compared to the supine position.
(4) Between the low and high physical activity level groups, there were significant differences in the abdominal and thoracic breathing movement ratios (AB/TB) in the sitting, standing, and supine positions. However, the respiratory rate (RR) differed only in the sitting position.

These findings may help improve the efficacy of breathing not only in healthy individuals but also in those with respiratory diseases, as well as during increased physical activity, exercise, and sports.

## Figures and Tables

**Table 1:** Demographic and anthropometric data of the participants (n = 37): n: number of subjects; BMI: body mass index; BSA: body surface area; SBP and DBP: systolic and diastolic blood pressure; HR: heart rate; low PA group h/week: low physical activity level group and their hours of activity level/week; high PA group h/week: high physical activity level and their hours of activity level/week; AB inspiration: abdominal circumference in centimeters during maximal inhalation; AB expiration: abdominal circumference in centimeters during maximal exhalation; TB inspiration: chest/thoracic circumference in centimeters during maximal inhalation; TB expiration: chest/thoracic circumference in centimeters during maximal exhalation.

	%	Mean ± SD
**Demographic and anthropometric data**		
Female/Male	48.6/51.4	
Age, year		21.0 ± 2.0
Weight, kg		68.3 ± 12.2
Height, cm		177.2 ± 8.5
BMI, kg/m^2^		21.6 ± 2.6
BSA, m^2^		1.8 ± 0.2
**Hemodynamic data**		
SBP (mmHg)		126.8 ± 14.2
DBP (mmHg)		72.3 ± 9.0
HR (1/min)		77.5 ± 12.4
**Respiratory data**		
AB inspiration (cm)		79.5 ± 8.4
AB expiration (cm)		76.2 ± 8.4
TB inspiration (cm)		87.5 ± 8.2
TB expiration (cm)		79.9 ± 7.2
**Training data**		
Training hours/week (n37)		7.2 ± 3.2
Low PA group h/week (n15)		2.5 ± 1.9
High PA group h/week (n22)		9.3 ± 1.8

**Table 2:** Differences between the low and high PA groups in AB and TB (%) and RR (1/min) measured in each body position. AB, abdominal breathing; TB, thoracic breathing; *g*, Hedges’ g; *p*, significance value; *t*, *t*-value; SD, standard deviation.

		Low PA		High PA		*t*	*p*	*g*
		Mean	SD	Mean	SD			
Sitting	AB	39.1	10.6	49.8	14.7	2.539	0.016	0.78
	TB	60.9		50.3				
Standing	AB	33.5	12.8	45.3	20.3	2.176	0.036	0.65
	TB	66.5		54.7				
Supine	AB	45.8	11.2	64.2	16.2	4.083	<0.001	1.25
	TB	54.2		35.8				
Prone	AB	49.0	24.2	54.4	22.6	0.687	0.498	0.22
	TB	51.0		45.6				
RR sitting		16.8	3.6	14.0	3.8	2.216	0.034	0.72
RR standing		16.3	3.6	14.2	3.9	1.685	0.102	0.54
RR supine		14.4	3.4	13.3	3.9	0.887	0.381	0.28
RR prone		15.6	4.9	13.7	4.1	1.224	0.232	0.42

## Data Availability

Data will be made available upon reasonable request.
